# Disruption of the sea turtle magnetic map sense by a magnetic pulse

**DOI:** 10.1242/jeb.251243

**Published:** 2025-11-20

**Authors:** Alayna G. Mackiewicz, Abigail M. Glazener, Kayla M. Goforth, Dana S. Lim, Catherine M. F. Lohmann, Kenneth J. Lohmann

**Affiliations:** Department of Biology, University of North Carolina at Chapel Hill, Chapel Hill, NC 27516, USA

**Keywords:** Map sense, Magnetoreception, Turtles, Magnetic pulse, Magnetite, Navigation

## Abstract

Although some migratory animals can derive directional (compass) and positional (map) information from Earth's magnetic field, the underlying mechanisms of magnetic sensing have remained enigmatic. One hypothesis proposes that crystals of the mineral magnetite (Fe_3_O_4_) function in magnetoreception, a concept bolstered by findings that brief, strong magnetic pulses capable of reversing the magnetic dipole moment of magnetite affect magnetic orientation responses of several animals. Disentangling whether such pulses affected an animal's magnetic compass sense or magnetic map sense, however, has often been difficult. Here, we investigated the effect of a magnetic pulse on the magnetic map sense of loggerhead sea turtles (*Caretta caretta*) using an established conditioning assay that requires turtles to use magnetic map information but not their magnetic compass. We report that a magnetic pulse disrupted turtle responses, consistent with the interpretation that the magnetic map sense of turtles is based at least partly on magnetite-based magnetoreceptors.

## Teaser

**Highlighted Article:** A brief, strong magnetic pulse disrupts responses of sea turtles to magnetic map cues, suggesting that the magnetic map sense of turtles relies on magnetite-based magnetoreceptors.

## INTRODUCTION

Magnetoreception – the ability to sense Earth's magnetic field – is widespread among animals (for reviews, see Begall et al., 2013; Wiltschko and Wiltschko, 1995, 2022; Naisbett-Jones and Lohmann, 2022). For species that migrate long distances, two different types of information useful in guiding movements can be derived from the geomagnetic field. Many animals have a magnetic compass sense, meaning that they use the geomagnetic field as a source of directional information that enables them to set and maintain headings towards, for example, north or south. In addition, several magnetic parameters, such as inclination angle (the angle at which field lines intersect the Earth's surface) and intensity (the strength of the field), vary predictably across the globe (Lohmann et al., 2022). The specific magnetic signatures (combinations of magnetic field parameters) that exist at different locations are used by sea turtles and other animals as a kind of ‘magnetic map’ for assessing geographic location (Lohmann et al., 2004, 2012; Putman et al., 2011, 2013; Wynn et al., 2020; Goforth et al., 2025).

Compelling evidence indicates that animals use geomagnetic cues in orientation and navigation, but how animals detect magnetic information has remained a long-standing mystery of sensory biology (Johnsen and Lohmann, 2005; Nordmann et al., 2017; Merlin, 2023; Vortman et al., 2025). Moreover, growing evidence suggests that, in some animals, different mechanisms underlie the magnetic compass and map, so that a single species may have two different ways of transducing magnetic signals to the nervous system (Lohmann, 2010; Wiltschko and Wiltschko, 2022; Phillips and Diego-Rasilla, 2022; Goforth et al., 2025).

Although a variety of biophysical mechanisms have been proposed for magnetic field detection, most research has focused on two hypotheses. The chemical magnetoreception hypothesis proposes that a series of light-initiated chemical reactions involving electron transfer and interactions of radicals are influenced by Earth-strength magnetic fields and might enable animals to detect directional (e.g. magnetic compass) information (Schulten et al., 1978; Rodgers and Hore, 2009; Ritz et al., 2009; Hore and Mouritsen, 2016). A leading hypothesis for the mechanism underlying the magnetic map sense posits that small particles of biogenic magnetite (Fe_3_O_4_) are a key part of the magnetoreceptor structure (Kirschvink et al., 2001; Shaw et al., 2015; Taylor et al., 2017). The simplest version of the magnetite hypothesis proposes that single-domain magnetite crystals rotate into alignment with Earth's magnetic field and, in so doing, activate receptors that initiate a neural response. One test for a magnetite-based mechanism involves pulse magnetization in which animals are exposed to a magnetic pulse, that is, a transient magnetic field strong enough to change the magnetic dipole moment of the crystals. The resulting disruption of the magnetite particle arrangement might impair the transduction of magnetic information to the nervous system. Importantly, a magnetic pulse might thus influence magnetite-based receptors but should not affect chemical magnetoreception (Wiltschko et al., 2002; Shaw et al., 2015).

Although the magnetic orientation responses of several species are altered by magnetic pulses (Wiltschko et al., 1994; Beason et al., 1997; Irwin and Lohmann, 2005; Holland, 2010; Ernst and Lohmann, 2016; Fitak et al., 2020; Naisbett-Jones et al., 2020), it has frequently been difficult to determine with confidence whether the animal's magnetic compass or its map sense was affected because the two are often used together in navigation (Wiltschko and Wiltschko, 2022). Recently, however, a behavioral assay was developed for loggerhead sea turtles (*Caretta caretta*), in which turtles use their map sense independently of their compass sense (Goforth et al., 2025). In this assay, individuals were conditioned to associate a specific magnetic signature with a food reward. After conditioning, turtles displayed higher amounts of food-anticipatory behavior (also referred to as the ‘turtle dance’) in the magnetic field in which they were fed relative to magnetic fields in which they were not. This assay was previously used to demonstrate that the map sense is unlikely to rely on chemical magnetoreception (Goforth et al., 2025). Here, we used the same map assay, in combination with magnetic pulses, to investigate whether a magnetite-based mechanism might serve as the basis of the turtles' map sense.

## MATERIALS AND METHODS

### Animal care and handling

Hatchling loggerhead turtles, *Caretta caretta* (Linnaeus, 1758) (*n*=16 total, with two drawn from each of eight nests), were obtained from Bald Head Island, NC, USA, in August 2021. Turtles were collected as they emerged naturally from their nests and were transported to the University of North Carolina at Chapel Hill, NC, USA. They were housed in individual tanks with recirculating artificial seawater, maintained at a salinity of 29–30 ppt and a temperature of about 25°C. Their diet in captivity consisted of a gel mixture of protein, spirulina, squid and Marine Cuisine^®^. Conditioning and subsequent experiments were conducted between January and April 2022. At the time of the magnetic pulse experiments, turtles were approximately 8 months old, with a straight carapace length ranging between 7.3 and 15.2 cm and mass between 76 and 560 g. All experiments and procedures were conducted according to protocols approved by the University of North Carolina Institutional Animal Care and Use Committee (protocol no. 23-216) and the North Carolina Wildlife Resources Commission (permit ST44).

### Map assay conditioning procedure

The map assay has been described in detail by Goforth et al. (2025). Conditioning was conducted inside a single-wrapped magnetic coil system, consisting of three 4-coil systems arranged orthogonally (Alldred and Scollar, 1967). The outer wraps of each coil measured 2.1 m on a side, while the two inner wraps measured 2.2 m on a side. In an adjacent control room, an independent power supply on a constant-current setting supplied current to each coil. A computer and relay system in the control room was used to switch on the power supplies. Because all treatments required generation of a magnetic field and use of the same computer, sounds associated with coil operation were identical during conditioning and all experimental trials. Moreover, a human listener in the coil room could not detect any sound coming from the control room when the coil system was activated.

**Fig. 1.**
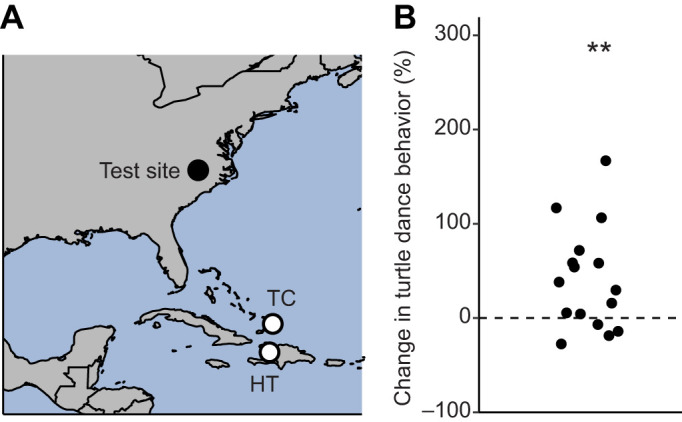
**Map assay experimental design and initial magnetic field discrimination following conditioning procedure.** (A) Map showing the locations of the test site (black circle); the magnetic signatures replicated magnetic fields that exist near Turks and Caicos (TC) and Haiti (HT), with the approximate locations indicated by white circles. (B) Percentage change in turtle dancing responses in the rewarded field relative to the unrewarded field for all conditioned turtles. The percentage change in turtle dance behavior was significantly greater than 0 (one-tailed Wilcoxon signed-rank test, *w*=116, *P*=5.49×10^−3^, *n*=16). The black dashed line represents a 0% change. Each dot represents the percentage change in dance behavior for an individual turtle. **P<0.01.

The coil system was used to produce magnetic signatures that matched two target areas in the Atlantic Ocean (Fig. 1A), one near Turks and Caicos and the other near Haiti (Table S1). These locations and the corresponding magnetic fields were similar to those used in a previous conditioning experiment (Goforth et al., 2025). Field parameters inside the coil were measured with a tri-axial magnetometer (Meda model FVM-400) in the area where turtles were positioned during conditioning and experimental trials (Table S1).

Eight of the turtles were conditioned over 2 months to associate food with the Turks and Caicos magnetic signature; thus, for this group, the Turks and Caicos field represented the rewarded field. The remaining eight turtles were rewarded with food in the Haiti magnetic signature. As in previous experiments (Goforth et al., 2025), each group of turtles was also exposed to an equal number of conditioning sessions in an unrewarded field; for the group fed in the Turks and Caicos field, the unrewarded field was the Haiti magnetic signature, and vice versa. Each day, turtles were placed inside the magnetic coil in individual buckets filled with artificial seawater. When the turtles were placed in the coil, the magnetic field was set to an ‘acclimation field’, which approximately replicated the magnetic field of the facility where the turtles are housed (Table S1). After 20 min, the field switched for 40 min either to the rewarded field (in which they received food) or to the unrewarded field (in which they did not). The next day, conditions were altered so that a turtle that had experienced the rewarded field the previous day now experienced the unrewarded field, and vice versa. If turtles were in their rewarded field, they received food 5–15 min after the field was changed. If turtles were in their unrewarded field, they received no food. This alternating pattern continued throughout the conditioning period.

### Map assay experimental trials

Following 2 months of daily conditioning, we used turtle dance responses to determine whether turtles could discriminate between the rewarded and unrewarded magnetic signatures in the absence of food. Turtle dance behavior is a distinctive pattern of movement displayed by captive turtles anticipating food. It includes some or all of the following: vertical tilt of the body, holding the head out of the water, opening the mouth and alternating front flipper movement (Goforth et al., 2025). On the day of testing, each turtle began the trial with a 20 min period in the acclimation field. The magnetic signature was then switched to the rewarded or unrewarded field for another 20 min; however, no food was provided. As turtles were fed every other day during the conditioning procedure, each turtle was tested only on days when it would have been fed in accordance with the alternating schedule. Trials were recorded using a GoPro (HERO7) camera positioned within the coil system and above the turtle's bucket. Each turtle was always tested at the same time of day to control for possible daily variation in activity levels. Water temperature was recorded before and after each trial to ensure that the temperature remained consistent throughout. At the end of each trial day, turtles that had been tested that day were fed in the rewarded field to reinforce the conditioned response.

Magnetic pulse experiments were undertaken approximately 3 weeks after the tests for conditioned field discrimination. The experiments were conducted within the 4 month period during which turtles are known to retain their conditioned responses even without reinforcement (Goforth et al., 2025), but to further ensure that responses were maintained, conditioning was continued on an every-other-day schedule until all experiments were completed.

### Magnetic pulse behavioral experiments

To generate a magnetic pulse, we used a magnetizer (Magnetic Instrumentation model 7515-G) consisting of a bank of capacitors that discharged to a solenoid (32 cm diameter×20 cm length) to produce a strong but brief magnetic field (duration 5 ms, maximum intensity 85 mT). The duration and intensity of this magnetic pulse were within the range used in previous studies involving pulse magnetization (Wiltschko et al., 1994, 1998, 2002; Munro et al., 1997; Beason et al., 1997; Holland, 2010), including several that used the same magnetizer (Irwin and Lohmann, 2005; Ernst and Lohmann, 2016; Naisbett-Jones et al., 2020; Ernst et al., 2020).

Turtles were tested after three different magnetic pulse treatments: (1) no pulse (a control in which turtles were not handled prior to testing); (2) sham pulse (a second control in which the turtle was placed into the pulse magnetizer, but a pulse was not applied); and (3) pulse (a brief, strong magnetic pulse was applied). Given that the effect of a magnetic pulse might hypothetically depend on the alignment of magnetite particles relative to the direction of the pulse (Wiltschko et al., 2002; Ernst and Lohmann, 2016), turtles received a magnetic pulse either parallel or antiparallel to the horizontal component of Earth's magnetic field (Ernst and Lohmann, 2016). Each turtle was randomly assigned to the parallel or anti-parallel pulse groups and only pulsed in that direction (parallel *n*=8, antiparallel *n*=8; see Fig. S2 for a schematic diagram). For the sham magnetic pulse treatment, turtles were handled in the same manner as in the magnetic pulse treatment, but the pulse was not applied. For both the sham-pulse and pulse treatments, turtles were subjected to the treatment the day before testing and then allowed to recover overnight (at least 12 h) before experimental trials were conducted the following day.

For all treatments, we recorded turtle dance behavior in the rewarded field. If pulse magnetization disrupted the map sense, we predicted that the responses of pulsed turtles would decrease compared with the no-pulse and sham-pulse treatments. Although previous studies indicate that the effects of magnetic pulses on animals are temporary (Wiltschko et al., 1994, 1998), the duration of potential effects in turtles is not known. To prevent the possibility that turtles in the two control treatments might be affected by a prior pulse treatment, turtles were first subjected to the no-pulse and sham-pulse treatments and then exposed to the pulse treatment. The order in which the turtles received the no-pulse and sham-pulse treatments was randomly assigned (i.e. half the turtles received the no-pulse treatment first, while the other half received the sham-pulse treatment first).

### Data analysis

The total time each turtle spent exhibiting turtle dance behavior was determined for the 20 min test period using BORIS (Behavioral Observation Research Interactive Software; Friard and Gamba, 2016). All videos were analyzed by two observers blind to the treatment. Before analyses, each observer was trained to recognize turtle dance behavior. The duration of time spent exhibiting this behavior was averaged across both observations for each trial (Tables S2 and S3).

All statistical tests and graphics were completed using R statistical software (version 2024.09.1; http://www.R-project.org/). To determine whether turtles had learned to discriminate between their rewarded and unrewarded field following the initial conditioning procedure, the percentage change in turtle dancing behavior in the rewarded field was calculated relative to the unrewarded field for each individual and was defined as:
(1)

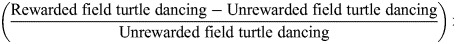
Given our *a priori* prediction that turtles would exhibit higher levels of turtle dancing in the rewarded field than in the unrewarded field, we used a one-tailed Wilcoxon signed-rank test to determine whether the percentage change in turtle dance behavior was greater than zero (Fig. 1B). A two-tailed Wilcoxon signed-rank test was also used to compare the total amount of time spent turtle dancing in response to the rewarded and unrewarded fields (Fig. S1).

For the magnetic pulse behavioral experiments, the amount of time spent turtle dancing in the parallel and antiparallel pulse groups was compared using a two-tailed Wilcoxon signed-rank test. Given that no difference was detected in responses to the parallel and antiparallel treatments, the two were combined as one group for subsequent analyses (Fig. S2).

**Fig. 2.**
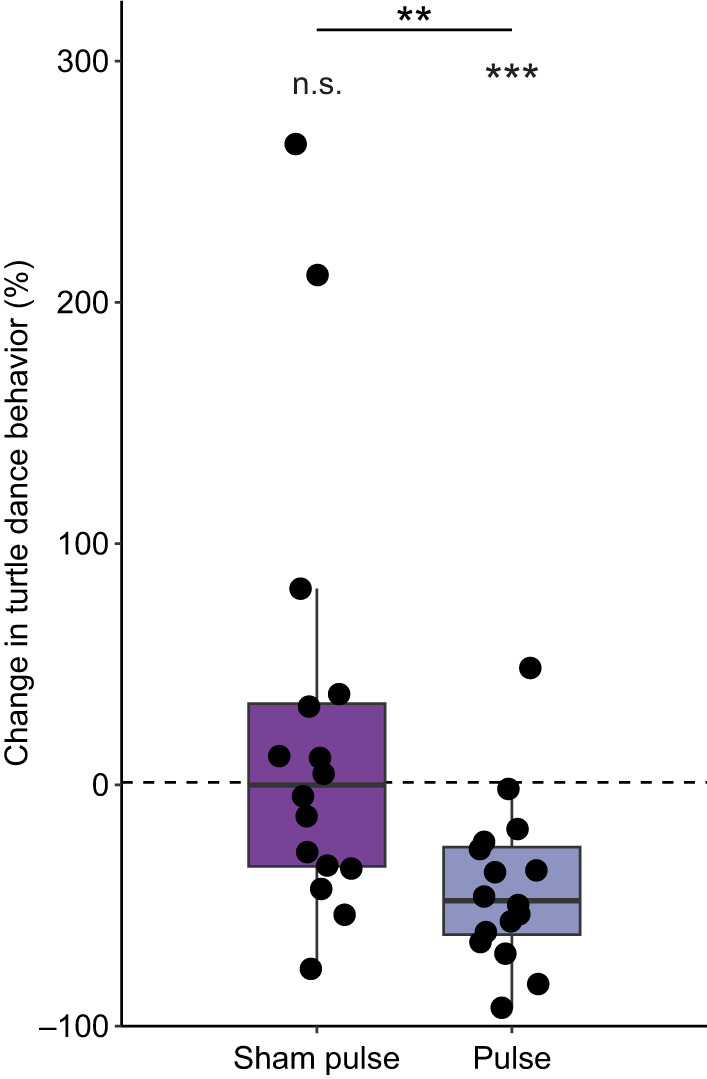
**The effects of magnetic pulse treatments on turtle dance behavior plotted relative to the control no-pulse treatment.** For the sham-pulse treatment, the percentage change relative to the control no-pulse treatment was not significantly less than 0 (one-tailed Wilcoxon signed-rank test, *w*=70, *P*=0.55, *n*=16). The percentage change for the pulse group was significantly less than 0 (one-tailed Wilcoxon signed-rank test, *w*=8, *P*=3.82×10^−4^, *n*=16). The percentage change for the sham-pulse and pulse treatments was significantly different (two-tailed Wilcoxon signed-rank test, *w*=120, *P*=5.16×10^−3^, *n*=16). Conventions as in Fig. 1B. Data are from all trials and turtles were tested in their rewarded fields. The boxes span the first and third quartiles, the center line represents the median, and the whiskers represent the 5th and 95th percentiles. n.s., not significant; **P<0.01, ***P<0.001.

We next calculated the percentage change in turtle dancing behavior in the rewarded magnetic signature for the sham-pulse and pulse treatments relative to the no-pulse treatment. Percentage change in turtle dance behavior was defined as:
(2)

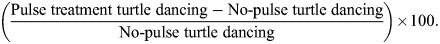
We predicted that if the sham-pulse or pulse treatment had an effect, then turtles should exhibit less turtle dance behavior compared with the no-pulse treatment (i.e. the percentage change in turtle dance behavior would be less than zero). Given this, we used a one-tailed Wilcoxon signed-rank test to determine whether the levels of turtle dance behavior in the sham-pulse and pulse treatments were significantly less than those for the no-pulse treatment (Fig. 2). Differences in percentage change for the sham-pulse and pulse treatments were compared using a two-tailed Wilcoxon signed-rank test (Fig. 2).

In a complementary analysis, we used a Kruskal–Wallis test to compare the total amount of time spent exhibiting turtle dance behavior across no-pulse, sham-pulse and pulse treatments as a whole. Pairwise comparisons were then made using a two-tailed Wilcoxon signed-rank test with a Benjamini–Hochberg correction (Table S4, Fig. S3).

## RESULTS AND DISCUSSION

After conditioning, turtles exhibited significantly higher levels of turtle dance behavior when experiencing the field in which they had been fed. Specifically, the percentage of time that turtles spent dancing in the rewarded field, relative to that in the unrewarded field, was greater than zero (*P*=5.49×10^−3^; Fig. 1B). Analysis of total time spent dancing also indicated that turtles responded more to the rewarded field than to the unrewarded field (*P*=0.04; Fig. S1). These findings are consistent with those obtained in a previous study (Goforth et al., 2025) and demonstrate that turtles can learn to associate food with a particular magnetic signature, providing the foundation for subsequent experiments.

We next investigated levels of turtle dance behavior in the rewarded field after the three different magnetic pulse treatments (no pulse, sham pulse and pulse). To analyze individual turtle dance behavior in the pulse treatments, we calculated the percentage change of sham-pulse and pulse treatments relative to the no-pulse treatment. The percentage change for the sham-pulse treatment was not significantly less than zero, indicating that the handling process, in the absence of a magnetic pulse, did not affect turtle behavior (*P*=0.55; Fig. 2). By contrast, the percentage change for turtles in the pulse treatment was significantly less than zero, indicating that the pulse resulted in turtles spending less time exhibiting turtle dance behavior (*P*=3.82×10^−4^; Fig. 2). Notably, the percentage change for the sham-pulse treatment and that for the pulse treatment were significantly different (*P*=5.16×10^−3^; Fig. 2). Overall, total time spent dancing also differed significantly among the three treatments (*P*=0.005; Fig. S3; see Table S4 for pairwise comparisons).

The results indicate that a brief, strong magnetic pulse reduced the responses of turtles to a magnetic signature that they had learned to associate with food. In contrast, the sham-pulse treatment, in which turtles were handled identically but not exposed to a magnetic pulse, did not result in any such change. The conditioned task required turtles to use their magnetic map sense to distinguish between two magnetic fields, without requiring the use of the magnetic compass sense (Goforth et al., 2025). Thus, the findings are consistent with the interpretation that magnetite-based magnetoreceptors underlie the map sense of turtles, inasmuch as a magnetic pulse can potentially disrupt such receptors (Williamson et al., 1983; Kirschvink et al., 2001; Johnsen and Lohmann, 2005), but is not expected to affect other proposed transduction mechanisms (Wiltschko et al., 2002; Shaw et al., 2015; Naisbett-Jones et al., 2020).

Previous work (Goforth et al., 2025) indicated that the turtle magnetic map sense is unlikely to be based on chemical magnetoreception, but provided no further insight into the likely mechanism. Using the same conditioning techniques employed in our current study, the earlier work demonstrated that the ability to discriminate magnetic signatures was unaffected by broadband oscillating magnetic fields in the radiofrequency range, a treatment expected to disrupt radical-pair-based chemical magnetoreception (Ritz et al., 2000; Henbest et al., 2004). Interestingly, similar radiofrequency fields disrupted the orientation of hatchling turtles in a different assay that required simultaneous use of both the magnetic compass and map (Goforth et al., 2025), suggesting that the compass, unlike the map, is based on chemical magnetoreception. Our new results build upon the previous study by demonstrating that a magnetic pulse disrupted the magnetic map sense, an outcome that should not have occurred if the map sense were based on chemical magnetoreception; thus, the new findings provide additional evidence for dual magnetoreception systems in turtles.

The findings in turtles are also consistent with studies on migratory birds, which may also have a magnetic compass sense involving chemical magnetoreception and a magnetic map sense relying at least partly on a magnetite-based mechanism (reviewed in Wiltschko and Wiltschko, 2022; Karwinkel et al., 2024). In birds, individuals migrating for the first time are thought to be guided by a compass sense alone, whereas experienced individuals are thought to acquire and use a map sense along with the compass sense (Munro et al., 1997). Notably, in a study with Australian silvereyes (*Zosterops lateralis*), a magnetic pulse did not disrupt the orientation of naive juvenile birds but did affect the orientation of experienced adult birds that had completed at least one migration, suggesting that the effect was on a magnetic map sense (Wiltschko et al., 1994, 1998; Munro et al., 1997). Furthermore, radiofrequency fields have been shown to affect the orientation responses of juvenile birds, presumably by disrupting the magnetic compass (Wiltschko et al., 2015).

Although pulse magnetization is considered by some to be a diagnostic test for magnetite-based magnetoreception (Kirschvink et al., 2001), the possibility of non-specific effects must also be considered. In fish and lobsters, for example, multiple genes were differentially expressed after a magnetic pulse (Fitak et al., 2017; Ernst et al., 2020). Some of these genes were linked to iron binding and regulation, but not all were, a result that raises the possibility that magnetic pulses affect processes unrelated to magnetoreception (Ernst et al., 2020). We did not observe any evident effects of magnetic pulses on health, motivation or activity levels, nor, to our knowledge, have such effects been reported in other animals; nevertheless, such effects cannot be entirely excluded. Additionally, turtles might be affected by the transient electric field induced by a magnetic pulse, particularly if they rely on electromagnetic induction to sense magnetic fields (Nimpf et al., 2019), and if the impact of a transitory electric field persists for at least 12 h (the time between the magnetic pulse and experimental trials).

In summary, our overall findings are consistent with the interpretation that a magnetite-based mechanism underlies the magnetic map sense in turtles, yet we caution that alternative explanations cannot be entirely ruled out. Because the assay we used is the first in any animal to decouple the magnetic map from the magnetic compass, the study provides the most direct evidence to date that a magnetic pulse affects the map sense as opposed to a magnetic compass. These results, combined with other recent findings (Goforth et al., 2025), bolster growing evidence that two different mechanisms of magnetoreception underlie the magnetic compass and map in some animals. Whether dual magnetoreceptor systems are widespread in the animal kingdom, or are restricted to a few animals that migrate long distances, awaits future investigation.

## Supplementary Material

10.1242/jexbio.251243_sup1 Supplementary information

## References

[JEB251243C1] Alldred, J. C. and Scollar, I. (1967). Square cross section coils for the production of uniform magnetic fields. *J. Sci. Instrum.* 44, 775. 10.1088/0950-7671/44/9/327

[JEB251243C2] Beason, R. C., Wiltschko, R. and Wiltschko, W. (1997). Pigeon homing: effects of magnetic pulses on initial orientation. *Auk* 114, 405-415. 10.2307/4089242

[JEB251243C3] Begall, S., Malkemper, E. P., Červený, J., Němec, P. and Burda, H. (2013). Magnetic alignment in mammals and other animals. *Mamm. Biol.* 78, 10-20. 10.1016/j.mambio.2012.05.005

[JEB251243C4] Ernst, D. A. and Lohmann, K. J. (2016). Effect of magnetic pulses on Caribbean spiny lobsters: implications for magnetoreception. *J. Exp. Biol.* 219, 1827-1832. 10.1242/jeb.136036 27045095

[JEB251243C5] Ernst, D. A., Fitak, R. R., Schmidt, M., Derby, C. D., Johnsen, S. and Lohmann, K. J. (2020). Pulse magnetization elicits differential gene expression in the central nervous system of the Caribbean spiny lobster, *Panulirus argus*. *J. Comp. Physiol. A* 206, 725-742. 10.1007/s00359-020-01433-7 32607762

[JEB251243C6] Fitak, R. R., Wheeler, B. R., Ernst, D. A., Lohmann, K. J. and Johnsen, S. (2017). Candidate genes mediating magnetoreception in rainbow trout (*Oncorhynchus mykiss*). *Biol. Lett.* 13, 20170142. 10.1098/rsbl.2017.0142 28446619 PMC5414700

[JEB251243C7] Fitak, R. R., Wheeler, B. R. and Johnsen, S. (2020). Effect of a magnetic pulse on orientation behavior in rainbow trout (*Oncorhynchus mykiss*). *Behav. Processes* 172, 104058. 10.1016/j.beproc.2020.104058 31954808

[JEB251243C8] Friard, O. and Gamba, M. (2016). BORIS: a free, versatile open-source event-logging software for video/audio coding and live observations. *Methods Ecol. Evol.* 7, 1325-1330. 10.1111/2041-210X.12584

[JEB251243C9] Goforth, K. M., Lohmann, C. M. F., Gavin, A., Henning, R., Harvey, A., Hinton, T. L., Lim, D. S. and Lohmann, K. J. (2025). Learned magnetic map cues and two mechanisms of magnetoreception in turtles. *Nature* 638, 1015-1022. 10.1038/s41586-024-08554-y 39939776

[JEB251243C10] Henbest, K. B., Kukura, P., Rodgers, C. T., Hore, P. J. and Timmel, C. R. (2004). Radio frequency magnetic field effects on a radical recombination reaction: a diagnostic test for the radical pair mechanism. *J. Am. Chem. Soc.* 126, 8102-8103. 10.1021/ja048220q 15225036

[JEB251243C11] Holland, R. A. (2010). Differential effects of magnetic pulses on the orientation of naturally migrating birds. *J. R. Soc. Interface* 7, 1617-1625. 10.1098/rsif.2010.0159 20453067 PMC2988258

[JEB251243C12] Hore, P. J. and Mouritsen, H. (2016). The radical-pair mechanism of magnetoreception. *Annu. Rev. Biophys.* 45, 299-344. 10.1146/annurev-biophys-032116-094545 27216936

[JEB251243C13] Irwin, W. P. and Lohmann, K. J. (2005). Disruption of magnetic orientation in hatchling loggerhead sea turtles by pulsed magnetic fields. *J. Comp. Physiol. A* 191, 475-480. 10.1007/s00359-005-0609-9 15765235

[JEB251243C14] Johnsen, S. and Lohmann, K. J. (2005). The physics and neurobiology of magnetoreception. *Nat. Rev. Neurosci.* 6, 703-712. 10.1038/nrn1745 16100517

[JEB251243C15] Karwinkel, T., Peter, A., Holland, R. A., Thorup, K., Bairlein, F. and Schmaljohann, H. (2024). A conceptual framework on the role of magnetic cues in songbird migration ecology. *Biol. Rev. Camb. Philos. Soc.* 99, 1576-1593. 10.1111/brv.13082 38629349

[JEB251243C16] Kirschvink, J. L., Walker, M. M. and Diebel, C. E. (2001). Magnetite-based magnetoreception. *Curr. Opin. Neurobiol.* 11, 462-467. 10.1016/S0959-4388(00)00235-X 11502393

[JEB251243C17] Lohmann, K. J. (2010). Q&A: animal behaviour: magnetic-field perception. *Nature* 464, 1140-1142. 10.1038/4641140a 20414302

[JEB251243C18] Lohmann, K. J., Lohmann, C. M. F., Ehrhart, L. M., Bagley, D. A. and Swing, T. (2004). Animal behaviour: geomagnetic map used in sea-turtle navigation. *Nature* 428, 909-910. 10.1038/428909a 15118716

[JEB251243C19] Lohmann, K. J., Putman, N. F. and Lohmann, C. M. F. (2012). The magnetic map of hatchling loggerhead sea turtles. *Curr. Opin. Neurobiol.* 22, 336-342. 10.1016/j.conb.2011.11.005 22137566

[JEB251243C20] Lohmann, K. J., Goforth, K. M., Mackiewicz, A. G., Lim, D. S. and Lohmann, C. M. F. (2022). Magnetic maps in animal navigation. *J. Comp. Physiol. A* 208, 41-67. 10.1007/s00359-021-01529-8 PMC8918461 34999936

[JEB251243C21] Merlin, C. (2023). Insect magnetoreception: a cry for mechanistic insights. *J. Comp. Physiol. A* 209, 785-792. 10.1007/s00359-023-01636-8 37184693

[JEB251243C22] Munro, U., Munro, J. A., Phillips, J. B., Wiltschko, R. and Wiltschko, W. (1997). Evidence for a magnetite-based navigational “map” in birds. *Naturwissenschaften* 84, 26-28. 10.1007/s001140050343

[JEB251243C23] Naisbett-Jones, L. C. and Lohmann, K. J. (2022). Magnetoreception and magnetic navigation in fishes: a half century of discovery. *J. Comp. Physiol. A* 208, 19-40. 10.1007/s00359-021-01527-w 35031832

[JEB251243C24] Naisbett-Jones, L. C., Putman, N. F., Scanlan, M. M., Noakes, D. L. G. and Lohmann, K. J. (2020). Magnetoreception in fishes: the effect of magnetic pulses on orientation of juvenile Pacific salmon. *J. Exp. Biol.* 223, jeb222091. 10.1242/jeb.222091 32291321

[JEB251243C25] Nimpf, S., Nordmann, G. C., Kagerbauer, D., Malkemper, E. P., Landler, L., Papadaki-Anastasopoulou, A., Ushakova, L., Wenninger-Weinzierl, A., Novatchkova, M., Vincent, P. et al. (2019). A putative mechanism for magnetoreception by electromagnetic induction in the pigeon inner ear. *Curr. Biol.* 29, 4052-4059. 10.1016/j.cub.2019.09.048 31735675

[JEB251243C26] Nordmann, G. C., Hochstoeger, T. and Keays, D. A. (2017). Magnetoreception – A sense without a receptor. *PLoS Biol.* 15, e2003234. 10.1371/journal.pbio.2003234 29059181 PMC5695626

[JEB251243C27] Phillips, J. B. and Diego-Rasilla, F. J. (2022). The amphibian magnetic sense(s). *J. Comp. Physiol. A* 208, 723-742. 10.1007/s00359-022-01584-9 36269404

[JEB251243C28] Putman, N. F., Endres, C. S., Lohmann, C. M. F. and Lohmann, K. J. (2011). Longitude perception and bicoordinate magnetic maps in sea turtles. *Curr. Biol.* 21, 463-466. 10.1016/j.cub.2011.01.057 21353561

[JEB251243C29] Putman, N. F., Lohmann, K. J., Putman, E. M., Quinn, T. P., Klimley, A. P. and Noakes, D. L. G. (2013). Evidence for geomagnetic imprinting as a homing mechanism in Pacific salmon. *Curr. Biol.* 23, 312-316. 10.1016/j.cub.2012.12.041 23394828

[JEB251243C30] Ritz, T., Adem, S. and Schulten, K. (2000). A model for photoreceptor-based magnetoreception in birds. *Biophys. J.* 78, 707-718. 10.1016/S0006-3495(00)76629-X 10653784 PMC1300674

[JEB251243C31] Ritz, T., Wiltschko, R., Hore, P. J., Rodgers, C. T., Stapput, K., Thalau, P., Timmel, C. R. and Wiltschko, W. (2009). Magnetic compass of birds is based on a molecule with optimal directional sensitivity. *Biophys. J.* 96, 3451-3457. 10.1016/j.bpj.2008.11.072 19383488 PMC2718301

[JEB251243C32] Rodgers, C. T. and Hore, P. J. (2009). Chemical magnetoreception in birds: The radical pair mechanism. *Proc. Natl. Acad. Sci. USA* 106, 353-360. 10.1073/pnas.0711968106 19129499 PMC2626707

[JEB251243C33] Schulten, K., Swenberg, C. E. and Weller, A. (1978). A biomagnetic sensory mechanism based on magnetic field modulated coherent electron spin motion. *Z. Phys. Chem.* 111, 1-5. 10.1524/zpch.1978.111.1.001

[JEB251243C34] Shaw, J., Boyd, A., House, M., Woodward, R., Mathes, F., Cowin, G., Saunders, M. and Baer, B. (2015). Magnetic particle-mediated magnetoreception. *J. R. Soc. Interface* 12, 0499. 10.1098/rsif.2015.0499 26333810 PMC4614459

[JEB251243C35] Taylor, B. K., Johnsen, S. and Lohmann, K. J. (2017). Detection of magnetic field properties using distributed sensing: a computational neuroscience approach. *Bioinspir. Biomim.* 12, 036013. 10.1088/1748-3190/aa6ccd 28524068

[JEB251243C36] Vortman, Y., Fitak, R. and Natan, E. (2025). Magnetoreception and the ruling hypothesis. *J. Exp. Biol.* 228, jeb250252. 10.1242/jeb.250252 40207401

[JEB251243C37] Williamson, S. J., Romani, G.-L., Kaufman, L. and Modena, I. (eds) (1983). *Biomagnetism: an Interdisciplinary Approach*. Boston, MA: Springer US.

[JEB251243C38] Wiltschko, R. and Wiltschko, W. (1995). *Magnetic Orientation in Animals*. Berlin, Heidelberg: Springer Berlin Heidelberg.

[JEB251243C39] Wiltschko, R. and Wiltschko, W. (2022). The discovery of the use of magnetic navigational information. *J. Comp. Physiol. A Neuroethol. Sens. Neural Behav. Physiol.* 208, 9-18. 10.1007/s00359-021-01507-0 34476571 PMC8918449

[JEB251243C40] Wiltschko, W., Munro, U., Beason, R. C., Ford, H. and Wiltschko, R. (1994). A magnetic pulse leads to a temporary deflection in the orientation of migratory birds. *Experientia* 50, 697-700. 10.1007/BF01952877

[JEB251243C41] Wiltschko, W., Munro, U., Ford, H. and Wiltschko, R. (1998). Effect of a magnetic pulse on the orientation of silvereyes, *Zosterops l. lateralis*, during spring migration. *J. Exp. Biol.* 201, 3257-3261. 10.1242/jeb.201.23.3257 9808838

[JEB251243C42] Wiltschko, W., Munro, U., Wiltschko, R. and Kirschvink, J. L. (2002). Magnetite-based magnetoreception in birds: the effect of a biasing field and a pulse on migratory behavior. *J. Exp. Biol.* 205, 3031-3037. 10.1242/jeb.205.19.3031 12200406

[JEB251243C43] Wiltschko, R., Thalau, P., Gehring, D., Nießner, C., Ritz, T. and Wiltschko, W. (2015). Magnetoreception in birds: the effect of radio-frequency fields. *J. R. Soc. Interface* 12, 20141103. 10.1098/rsif.2014.1103 25540238 PMC4305412

[JEB251243C44] Wynn, J., Padget, O., Mouritsen, H., Perrins, C. and Guilford, T. (2020). Natal imprinting to the Earth's magnetic field in a pelagic seabird. *Curr. Biol.* 30, 2869-2873. 10.1016/j.cub.2020.05.039 32559442

