# Supplementary material for: Disruption of the sea turtle magnetic map sense by a magnetic pulse
Source: J Exp Biol. 2025 Nov 20;228(22):jeb251243. doi: 10.1242/jeb.251243 (PMC12669835; doi:10.1242/jeb.251243)
Supplement: Supplementary information [file jexbio-228-251243-s1.pdf]

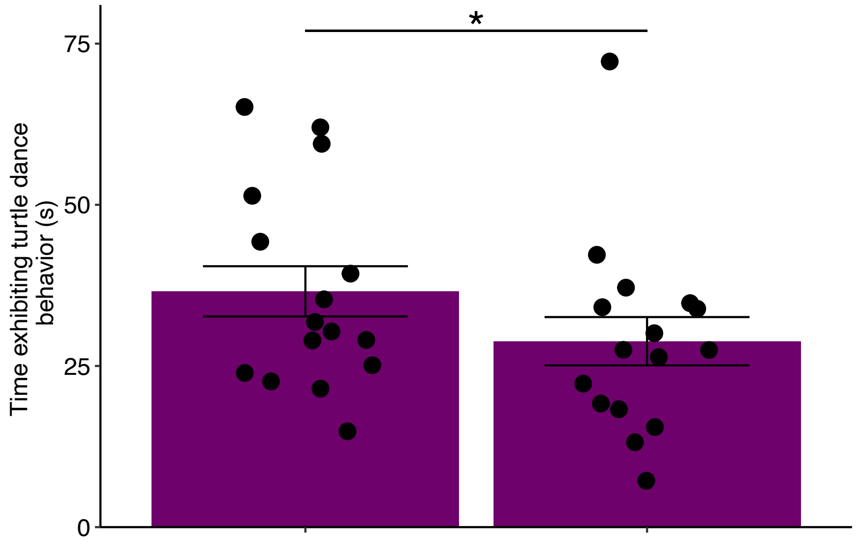

Fig. S1. Amount of time turtles spent dancing in the rewarded and unrewarded magnetic field. Turtles spent significantly more time exhibiting turtle dance behavior in the magnetic field in which they received food (the rewarded field) than in the magnetic field in which they did not (the unrewarded field) (two-tailed Wilcoxon signed-rank test, $w = 108$, $p = 0.04$, $n = 16$). Each dot represents results for one individual; error bars represent standard error.

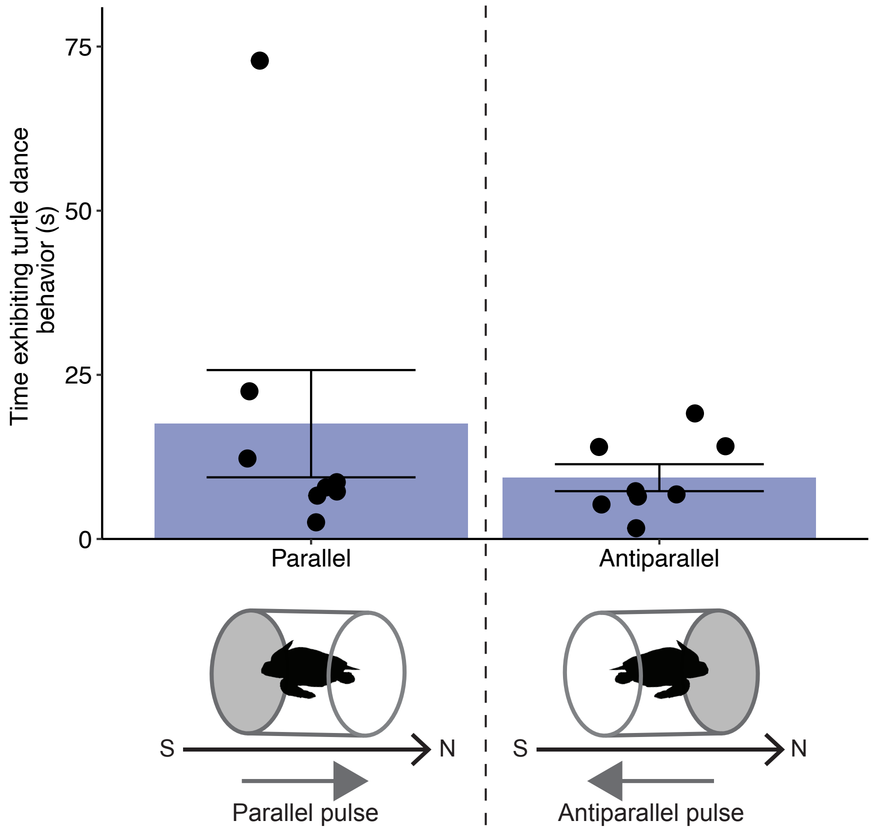

Fig. S2. Amount of time turtles spent dancing in the rewarded magnetic field after experiencing either a parallel or antiparallel magnetic pulse. There was no significant difference in responses between the two pulse groups (two-tailed Wilcoxon signed-rank test, $w = 39$, $p = 0.51$, $n = 8$). Each dot represents results for one individual; error bars represent standard error. The grey cylinder represents the solenoid with the top arrow (dark grey) denoting the direction of the magnetic pulse and the bottom arrow (black) representing the direction of magnetic north (N). Diagram of solenoid and turtle is not to scale.

Journal of Experimental Biology • Supplementary information

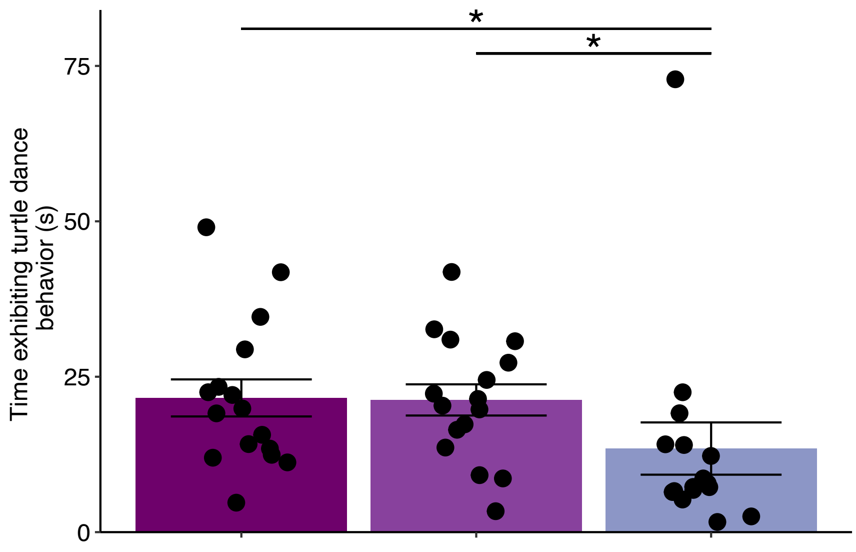

Fig. S3. Amount of time turtles spent dancing in the rewarded field after the no-pulse, sham-pulse, and pulse treatments. Analysis of total time spent dancing across all three treatments indicated a significant difference between treatments (Kruskal-Wallis, H = 10.739, df = 2, p = 0.005, n = 48). Pairwise comparison of no-pulse and sham-pulse treatments indicated no significant difference (two-tailed Wilcoxon signed-rank test with Benjamini-Hochberg correction, w = 73, p = 0.82, n = 16), suggesting that the handling process for the magnetic pulse had no impact on responses to the rewarded field. In contrast, turtles spent significantly less time dancing when tested in the rewarded field after receiving a pulse compared to their responses when not pulsed (two-tailed Wilcoxon signed-rank test with Benjamini-Hochberg correction, w = 122, p = 0.01, n = 16) and compared to their responses when sham-pulsed (two-tailed Wilcoxon signed-rank test with Benjamini-Hochberg correction, w = 116, p = 0.02, n = 16). Each dot represents results for one individual; error bars represent standard error.

Journal of Experimental Biology • Supplementary information

Table S1. Magnetic signatures used to approximate conditions in different geographic areas for the map assay. Values indicate the average of 8 measurements made using a tri-axial magnetometer (Meda model FVM-400) in the area within the coil where turtles were positioned during conditioning and experimental trials. Magnetic field estimates for locations near the Turks and Caicos and near Haiti were obtained from the International Geomagnetic Reference Field (IGRF) model 13 for fall 2021. The acclimation magnetic field mimicked the field of the turtle husbandry facility and differed slightly from the natural ambient magnetic field. Declination (difference between magnetic north and geomagnetic north) was held constant in all magnetic fields).

Magnetic field	Inclination (°)	Intensity (μT)	Latitude/longitude for field estimate
Turks and Caicos	48.5	39.7	21.8 N, -72.2 W
Haiti	45.7	37.9	19.2 N, -71.5 W
Acclimation	60.8	47.0	Not applicable

Table S2. Turtle dance data for initial magnetic field discrimination following the conditioning procedure.

Turtle ID	Treatment	Average turtle dance duration (s)
L217	Rewarded	22.60
L217	Unrewarded	13.16
L218	Rewarded	51.38
L218	Unrewarded	37.15
L219	Rewarded	29.06
L219	Unrewarded	27.50
L220	Rewarded	59.45
L220	Unrewarded	22.27
L221	Rewarded	44.30
L221	Unrewarded	34.12
L222	Rewarded	35.35
L222	Unrewarded	33.89
L223	Rewarded	21.52
L223	Unrewarded	26.40
L224	Rewarded	25.15
L224	Unrewarded	34.72
L225	Rewarded	62.02
L225	Unrewarded	72.24
L226	Rewarded	39.35
L226	Unrewarded	42.23
L227	Rewarded	23.91
L227	Unrewarded	15.51
L228	Rewarded	65.15
L228	Unrewarded	30.06
L229	Rewarded	30.38
L229	Unrewarded	19.20
L230	Rewarded	14.84
L230	Unrewarded	7.19
L231	Rewarded	28.98
L231	Unrewarded	18.29
L232	Rewarded	31.82
L232	Unrewarded	27.50

Table S3. Turtle dance data following the no pulse, sham pulse, and pulse (parallel or antiparallel) treatments.

Turtle ID	Pulse Treatment	Average turtle dance duration (s)
L217	No Pulse	29.40
L217	Parallel	22.50
L217	Sham Pulse	30.72
L218	No Pulse	11.22
L218	Parallel	7.24
L218	Sham Pulse	20.35
L219	Antiparallel	14.13
L219	No Pulse	22.07
L219	Sham Pulse	24.48
L220	Antiparallel	7.27
L220	No Pulse	41.81
L220	Sham Pulse	27.26
L221	No Pulse	15.66
L221	Parallel	7.85
L221	Sham Pulse	13.61
L222	Antiparallel	6.79
L222	No Pulse	22.51
L222	Sham Pulse	30.97
L223	Antiparallel	14.02
L223	No Pulse	19.11
L223	Sham Pulse	21.43
L224	No Pulse	14.16
L224	Parallel	6.61
L224	Sham Pulse	3.37
L225	No Pulse	49.04
L225	Parallel	72.85
L225	Sham Pulse	32.63
L226	Antiparallel	19.13
L226	No Pulse	23.37
L226	Sham Pulse	22.28
L227	No Pulse	34.63
L227	Parallel	2.53
L227	Sham Pulse	19.74
L228	Antiparallel	5.25
L228	No Pulse	13.44
L228	Sham Pulse	41.85

L229	Antiparallel	6.45
L229	No Pulse	11.97
L229	Sham Pulse	8.64
L230	No Pulse	19.89
L230	Parallel	8.64
L230	Sham Pulse	9.17
L231	Antiparallel	1.64
L231	No Pulse	4.74
L231	Sham Pulse	17.34
L232	No Pulse	12.44
L232	Parallel	12.26
L232	Sham Pulse	16.47

Table S4. Pairwise comparisons for pulse treatments (no pulse, sham pulse, and pulse).

Comparisons that yielded statistically significant differences are bolded.

Pulse treatment comparison	w	p-value	n
No pulse - Sham pulse	73	0.82	16
**No pulse - Pulse**	**122**	**0.01**	**16**
**Sham pulse - Pulse**	**116**	**0.02**	**16**
